# Contribution of Different Impairments to Restricted Knee Flexion during Gait in Individuals with Cerebral Palsy

**DOI:** 10.3390/jpm12101568

**Published:** 2022-09-23

**Authors:** Faustyna Manikowska, Sabina Brazevič, Marek Jóźwiak, Maria K. Lebiedowska

**Affiliations:** 1 Department of Pediatric Orthopedics and Traumatology, Poznań University of Medical Sciences, 61-701 Poznań, Poland; 2 Independent Researcher, Edmonton, AB T5R 2R2, Canada

**Keywords:** cerebral palsy, impairments, motor dysfunction, gait, motion analysis

## Abstract

The coexistence of overlapping impairments modulates the knee pattern in the swing phase of walking in children with cerebral palsy (CP). The impact and contribution of each impairment to the reduction of knee range-of-motion is unknown. The aim of the study was to establish the gradation of the impact of individual coexisting impairments on the knee flexion range-of-motion. Passive range-of-motion, selective motor control, strength, and spasticity from 132 patients (Male = 76, Female = 56, age:11 ± 4 years) with spastic CP were tested with clinical tools. Knee flexion range-of-motion at terminal stance, pre-swing, and initial swing phases were assessed by gait analysis. Hypertonia (β = −5.75) and weakness (β = 2.76) of knee extensors were associated with lower range of knee flexion (*R*^2^ = 0.0801, *F* = 11.0963, *p* < 0.0001). The predictive factors (*R*^2^ = 0.0744, *F* = 7.2135, *p* < 0.0001) were strength (β = 4.04) and spasticity (β = −2.74) of knee extensors and strength of hip flexors (β = −2.01); in swing those were knee extensors hypertonia (β = −2.55) and passive range of flexion (β = 0.16) (*R*^2^ = 0.0398, *F* = 3.4010, *p* = 0.01). Hypertonia of knee extensors has the strongest impact on knee flexion range-of-motion; secondary is the strength of knee extensors. The knee extensors strength with knee extensors hypertonia and strength of hip flexors contributes in stance. Knee extensors hypertonia with passive knee flexion range-of-motion contributes in swing.

## 1. Introduction

Children with motor disorders such as cerebral palsy (CP) presented a combination of clinical signs as consequences of upper motor neuron lesions. As an effect of brain damage, positive (increased involuntary muscle activity, movement, or movement pattern; e.g., hypertonia) and negative motor signs (insufficient muscle activity or its control; e.g., weakness, impaired selective muscle control) are observed. Multiple impairments may occur simultaneously and affect functional skills such as gait in CP individuals [1,2].

The knee range of motion (ROM) during a gait cycle (GC) in healthy individuals is usually around 60 deg. There usually exist two periods when the knee is flexed during GC. The first one occurs at the beginning of GC, and the second one starts in the middle of the terminal stance (TSt; ~40% of GC) and ends with knee maximum flexion in the middle of the initial swing (ISw; ~70% of GC). The first period of knee flexion occurs in the loading response phase and serves as a shock absorber and supports stability [3,4,5]. The second period of knee flexion is especially important as a major factor, besides terminal swing knee extension, affecting knee ROM during gait. The second period of knee flexion consists of two distinct periods: 1. flexion in stance: from the beginning of knee flexion at TSt to the toe off, increase from full knee extension to 40° of flexion; and 2. flexion in swing: increase from 40° to 60° of flexion in initial swing phase.

The gait efficiency, foot clearance, step length, and walking velocity are usually determined by proper knee flexion ROM, its velocity, and the timing of those events [4]. The decrease in ranges and velocity and delay of the peak of knee flexion was reported in a patient with CP [5,6,7].

The most common impairments (mainly hypertonia, weakness, and lack of selective motor control) affect the functional abilities of patients with CP. The selected impairments at different joints were previously reported as factors contributing to deviations in gait patterns. The weakness and/or hypertonia of knee extensors, hypertonia of knee flexors, hypertonia of plantar flexors, knee extensors contraction, limited thigh advancement caused by weakness of hip flexors or insufficient toe-off, and primitive synergies are all potential factors of pathological knee flexion in the swing phase of walking [4,5,8,9,10].

Previous studies usually focused on a single disregarding coexisting impairment. The effect of spasticity [10,11,12], inappropriate electrical firing activity of rectus femoris (RF) [6,7], RF muscle maximum length [8], growth and age [13], and decrease of range-of-motion (ROM) of knee flexion were recognized as factors decreasing ROM.

In addition, the most common orthopedic treatment surgeries are based on the same assumption of the significance of isolated factors. For example, RF transfer is supposed to improve the range and/or timing and velocity of knee flexion. The results of the treatment are not consistently successful [14,15,16,17]. Campbell et al. reported that the intervention is ineffective based on their meta-analysis [18]. Inconsistency in reported effectiveness seems to be dependent on the individual’s status. It was shown that this treatment is more beneficial in patients with predominant swing phase activity of RF and only those on higher functional status (GMFCS levels I & II) than those who are not independent walkers (GMFCS levels III & IV) [14,19]. It suggested that not only hypertonia, but weakness, lack of selective motor control, and contraction might affect the effectiveness of RF transfer surgery.

Thus, it seems that the coexistence of many overlapping impairments modulates the knee pattern in the swing phase of walking in patients with CP. However, the impact and contribution of each impairment to the reduction of knee ROM are unknown.

The present study aimed to establish the gradation of the impact of individual coexisting impairments on the knee flexion ROM. To achieve the aims of the study, the results on selected knee ranges of motion (three-dimensional gait analysis) and clinical tests (strength [MMT], hypertonia [HYP], selective motor control [SEL], and passive knee flexion ROM (CON) were analyzed.

## 2. Materials and Methods

In total, 132 patients (male, *n* = 76; female, *n* = 56; age: 11 ± 4 years) with spastic hypertonia were recruited for the study. Data from 262 limbs were analyzed; due to technical errors, data from two limbs were excluded. Data were collected from a local Gait and Motion Analysis Laboratory. To be enrolled in the study group, patients had to meet the inclusion criteria: complete data from clinical and gait analysis; no botulinum injection or surgery within six months before the assessment; be able to walk without physical assistance. The appropriate Institutional Review Board approved the study, and written consent for using the examination data was acquired from all participants. For participants under the age of 18, consent was obtained from a parent or legal guardian. Data from lower limb clinical examinations and gait analysis were investigated. Clinical examination, including passive range of motion, selective motor control, muscle strength, and muscle tone (around hip, knee, and ankle joints), were tested.

### 2.1. Protocols

Data from clinical examinations and gait analysis were investigated.

#### 2.1.1. Clinical Examination

- Passive knee flexion ROM: It was tested with a handheld goniometer; the passive angular value of movement was measured, and the presence or absence of impairment was documented according to the normal values [20];
- Selective motor control (SMC): SMC was graded from 0 (no ability to perform isolated movement) to 2 (presented movement is completely isolated). The presence of impairment was defined in limbs with SMC < 2; otherwise, the limbs were defined as not impaired [21];
- Manual muscle testing (MMT): MMT was graded from 0 (no evidence of muscle contraction) to 5 (ability to produce movement against the full external resistance). The presence of strength impairment was defined in limbs with a grade less than 4 [20];
- Muscle hypertonia (HYP): HYP was assessed with: Modified Ashworth Scale [22] and Tardieu Scale [23]. The presence of hypertonia was defined in limbs with a score higher than 0.

#### 2.1.2. Gait Analysis

An eight-camera three-dimensional gait analysis system (6 Bonita cameras and 2 Vero cameras; Vicon Motion Systems Ltd., Oxford, UK) sampling at 120 Hz was used. Reflective markers were applied according to the standard Plug-in-Gait marker placement model to each patient. Participants walked barefoot along a 10-m walkway at a self-selected speed. Pre-swing (PSw), and initial swing (ISw) phases of gait, the kinematic data were collected to measure ROM and velocity of knee flexion in the sagittal plane during terminal stance (TSt).

### 2.2. Data Analysis

#### 2.2.1. Outcome Measures

**Figure 1:**
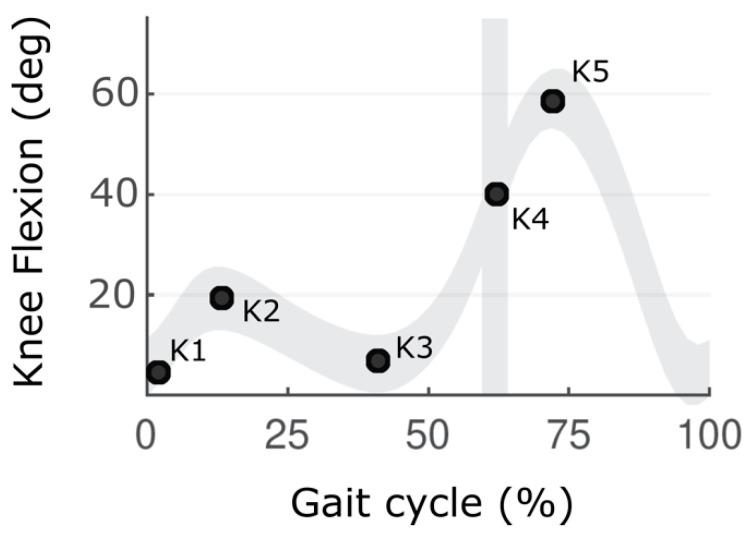
Knee kinematics in sagittal plane. [K1] Knee position at initial contact; [K2] maximum knee flexion at initial stance; [K3] Minimum knee position during terminal stance; [K4] Knee position at toe off; [K5] Maximum knee flexion during swing phase.

The primary outcome measures were selected ranges of knee motion in the sagittal plane during terminal stance (TSt), pre-swing (PSw), and initial swing (ISw). As shown in Figure 1, five key points in sagittal knee kinematics were identified in each GC for further analysis: [K1] Knee position at initial contact; [K2] In a normal pattern of gait, it refers to the maximum knee flexion at initial stance; in knee hyperextension, it indicates a minimum of knee extension in stance; [K3] Minimum knee position during terminal stance; [K4] Knee position at toe-off; and [K5] Maximum knee flexion during swing phase. Only K3, K4, and K5 were used in the present study. The ROM was considered as one value (from TSt to ISw) K3–K5 and also was divided into two parts: flexion in stance: K3–K4 and flexion in swing: K4–K5. In addition, knee flexion velocity at toe-off was included in the analysis.

Major factors that play a potential role in the biomechanics of knee joint flexion during TSt, PSw, and ISw phases were considered in the analysis. The hypertonia (hip and knee flexors, knee extensors, and ankle plantar flexors at slow and fast movement velocity), muscle strength (hip flexors and knee extensors), selective motor control (hip flexors and knee extensors), and restricted passive range of motion (knee extensors) were included in the analysis. The potential outcome measures within each impairment are listed in Table 1.

#### 2.2.2. Statistical Analysis

A multiple linear regression analysis was used to determine an impact. A multiple linear regression model was built to investigate the simultaneous effects of each of the outcome measures within selected impairments (independent variables) on the selected knee ROMs (dependent variable). It was assumed that *n* ≥ 50 + 8 k, where k is the number of explanatory variables in the model. The analysis of variance test (F test) was used to test the significance of the model. *T*-test was used to test the significance of individual parameters. The β coefficients were estimated using the classical least squares method. Statistical analysis was performed with the application of Statistica 13 (TIBCO Software Inc., Palo Alto, CA, USA), Stata (StataCorp LLC, College Station, TX, USA), and StatXact (Cytel Inc., Walthan, MA, USA) with a level of significance of *p* ≤ 0.05.

## 3. Results

### Multiple Regression Analysis (Gradation of Impact of Single Impairment When Coexisting of Multi-Impairments Were Stated)

Results of the multiple linear regression analysis showed that increased HYP_KNEE_EXT and reduced MMT_KNEE_EXT were associated with a lower range of knee flexion K5–K3. Moreover, HYP_KNEE_EXT was a twice stronger predictor than MMT_KNEE_EXT explained by the model.

Reduced range of knee flexion at initial (K5–K4) was associated with lower CON_KNEE_EXT and increased HYP_KNEE_EXT. Although two variables, SMC_HIP_FLEX and SMC_KNEE_EXT, did not show any significant association with K5–K4, removing them from the model would significantly reduce the adjusted *R*^2^. It must be noted that HYP_KNEE_EXT is the strongest predictor in this model. It is 16 times stronger than CON_KNEE_EXT.

MMT_KNEE_EXT, MMT_HIP_FL as well as HYP_KNEE_EXT were found to be significant predictive factors in the case of the K4–K3 range. An increase in HYP_KNEE_EXT, as well as MMT_HIP_FL, reduced the range of knee flexion during terminal stance (K4–K3). On the other hand, decreased MMT_KNEE_EXT, which is a twice stronger predictor than the other two in this model (MMT_HIP_FL and HYP_KNEE_EXT), will result in reduced K4–K3.

Increased knee angular velocity at toe-off was associated with a higher SMC_HIP_FLEX score and decreased HYP_RF. Both predictors revealed similar strengths in this model.

**Figure 2:**
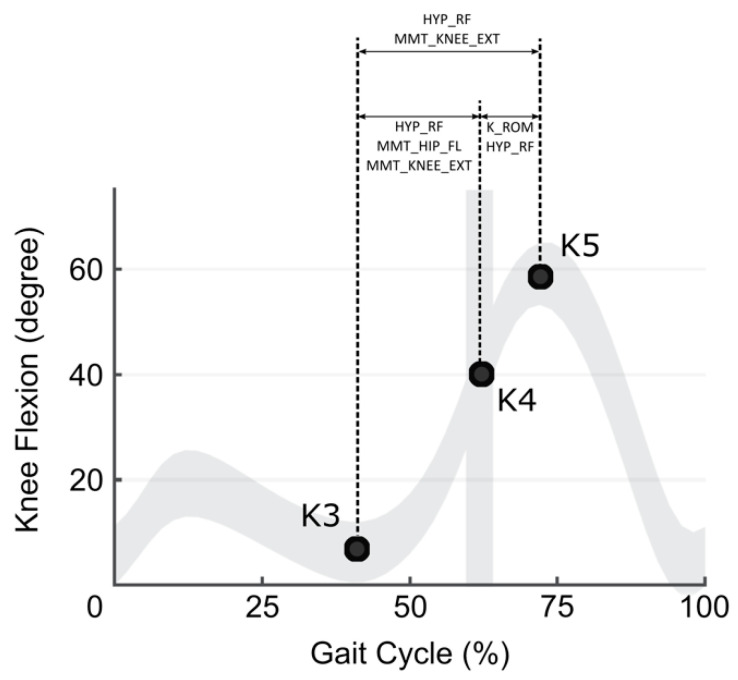
Contribution of impairments to knee kinematics during gait.

Neither HYP_PF nor HYP_KNEE_FL or SMC_KNEE_EXT were significantly associated with knee ROMs (Table 2, Figure 2).

## 4. Discussion

The present study aimed to establish the gradation of the impact of individual coexisting impairments on the selected knee flexion ranges of motion in the sagittal plane.

We found that the hypertonia of the knee extensor muscles had the most significant contribution (β = −5.75) to the restriction of knee flexion ROM from late stance to early swing K5–K3 (Figure 2; Table 2). The secondary factor that contributed to the increase of the K5–K3 range of flexion in those phases was the strength of knee extensor muscles (β = 2.76) (two times weaker than the contribution of hypertonia).

The knee flexion ROM during the stance phase from the minimum in TSt to the toe-off (K4–K3) depended strongest on the knee extensors strength (β = 4.04) and around 30% less than its hypertonia (β = −2.74) and 50% less than the strength of hip flexors (β = 2.01).

The knee flexion ROM between maximum and the beginning of swing (K5–K4) depended strongest on the knee extensors hypertonia (β = −2.55) and next on the knee flexion passive ROM (β = 0.16), knee extensors hypertonia was around 20 times stronger decreasing factor than passive ROM.

The knee flexion velocity at TSt/ISw was affected by knee extensor hypertonia (β = −39.86) and SMC of hip flexor with similar power (β = 39.68). The rest of investigated impairments did not show any effects on studied periods of knee flexion applying a developed statistical model of gradation of impairments.

We investigated the second period of knee flexion from the minimum in terminal stance and maximum in the swing phases of GC. The knee motion during this period affects the entire knee ROM and velocity of walking. During the entire period of knee flexion (K5–K3) and its parts (K4–K3) and (K5–K4), we found a substantial impact of RF hypertonia, strongest in K5–K3 and K5–K4. During this period, predominantly passive knee flexion takes place [4]. The participation of RF in these periods can be explained by the chain of biomechanical and neurophysiologic events. The predominantly passive knee flexion stretches the knee extensors (including RF) that might trigger spasticity (through exaggerated stretch reflexes) and the next increase in resistance to external stretch (spastic hypertonia). Thus, our data confirmed a potent contribution of knee extensor hypertonia on the restrictions of knee ROM leading the gait deterioration in ambulatory (GMCFS group I to IV) patients with CP. The role of RF hypertonia on knee flexion ROM is still debatable [24,25,26,27]. The origin of RF over activity is not clear, and the lack of standard, objective methods of hypertonia evaluation add some confusion. Some studies confirm its contribution, and some contradict it [24,25,26,27]. It was reported that different factors might affect knee flexion in the stance than in the swing phase based on an analysis of bioelectrical activation of RF muscle during gait. It was suggested that RF activity could decrease knee ROM during the pre-swing but not at the initial swing phase [26,27]. We found that RF hypertonia was the primary factor that affected knee flexion both in the in stance and in swing phases. However, we also found that in stance phase secondary and third, respectively, were the strength of hip flexors and strength of knee extensors. In swing, the secondary knee flexion ROM. It was previously widely discussed that hypertonia coexists with muscle weakness and impaired selective motor control [10,28,29,30,31,32]. Muscle strength can be affected by impaired neural function due to damage to the central nervous system and by muscle properties. Activation of muscles with proper reciprocal inhibition from antagonist during voluntary movement and/or improper motor unit recruitment might also be connected to spastic hypertonia [33,34]. Reduced selective muscle control is a concern, such as the impaired ability to produce isolated activation of the muscle. This occurs due to lesions in the descending spinal path but can also be affected by reflex irradiation and hypersensitivity to non-specific stimuli (e.g., sound) [1]. Thus, it can also be related to hypertonia.

It was previously reported that diminished knee flexion might be related to low knee flexion velocity at toe-off [12,35] because of the decreased push-off power caused by insufficient plantar flexors activity [4,15,36]. Our data agree that the lower velocity at push-off, the lower range of the motion. However, the knee velocity of the toe-off is inherently linked to the ROM (as a first-time derivate of motion). Our data suggested that the velocity of flexion in toe-off depends exactly to the same extent as knee extensors hypertonia as to the ability to produce selective hip flexion movement but is not related to the plantar-flexors hypertonia. Therefore, with the application of our statistical model, we could not confirm at this time an effect of plantar flexors hypertonia on knee flexion ROM.

It is generally accepted that hip flexors play an important role in walking. Walking with self-paced velocity usually does not require the significant activity of hip flexors because pendular leg motion is driven through the forward velocity of a body. Knee flexion in the swing phase is predominantly passive as a result of passive double pendulum leg movement that is propelled by forward body motion with a potential hip flexors activity and some contribution from push-off [4,5,37]. At a slower velocity of walking, typically acquired by patients with CP, the voluntary hip flexors activation might be necessary to replace the reduced dynamic drive [4]. Our finding proposed that in the stance (K3–K4), the primary contribution came from knee extensor strength, with secondary contribution from knee extensor hypertonia and strength of hip flexors confirmed by previous studies. We found that when the leg is in full swing (K5–K4), not only knee extensor hypertonia but also the passive range of knee flexion participated in the reduction of ROM.

The transfer or lengthening of the rectus femoris muscle is the most common orthopedic procedure to increase knee flexion during the swing phase of walking [38,39,40]. Theoretically, it was assumed that the transfer of RF and changing its function from knee extensor to flexion should increase the knee flexion ROM [39]; however, the mechanism and benefits of the surgery were recently seriously questioned [18,41,42]. Most likely, the lengthening of the RF and reducing knee extensors force led to the improvement in flexion [42]. Our data does not support this claim—it shows the positive influence of knee extensor strength on kinematics as a secondary predictor (with knee extensor hypertonia as a major factor). It was reported that RF surgery improves gait parameters predominantly in patients of GMFCS levels I and II but not in patients of GMFCS levels III and IV [14]. The more advanced the GMFCS level the more overlapping impairments not only restricted to muscles or joints might be responsible for this [43]. In the future, it seems reasonable to analyze the significance of coexisting impairments separately for CP independent and assisted walkers.

Our study provides the first quantitative data supporting not only an impact but also the gradation of the impact of multiple impairments on knee flexion during walking. Our data also offers strong confirmation of an effect of rectus femoris muscle biomechanical and neurophysiological properties on knee flexion in terminal stance and initial swing. In general, the primary effect is apparent through the muscle spastic hypertonia and a secondary effect through weakness of knee extensors and hip flexors and impairment of SMC of hip flexors.

### Limitations

The main limitation of the study was that we investigated data from all CP subjects as one study group putting aside the fact that patients with different levels of involvement presented different levels of severity in symptoms. Patients walking with aids were once weaker and more spastic, with the presence of more impairments than independent walkers [43].

Our study was based on the analysis of the presence or absence of specific impairment based on the results of clinical tests. Taking the low reliability of clinical measures into consideration, we reduced the results of the original tests into two categories. It is expected that the objective, quantitative measures within impairment could increase our understanding of the complex interactions between function and overlapping impairments.

In sum, the very strong contribution of RF hypertonia through the entire period of knee flexion and its parts was accompanied by the weaker contribution of the strength of knee extensors (K5–K3, K4–K3), hip flexors (K4–K3), passive knee range of the motion (K5–K4), and SMC of hip flexors (knee velocity at toe-off). The relationship between clinical parameters and GC is not easy to be established because of the different nature of this data, which is a result of the complex relationship between anatomical, clinical, biomechanical, and physiological factors [27,44,45]. Our data support the claim that, considering all present impairments, their severity and interaction potentially could customize the treatments to an individual’s needs.

## 5. Conclusions

Hypertonia of knee extensors has the primary impact on knee flexion ROM. The strength of knee extensors at the terminal stance up maximum in the swing has a secondary impact. In the stance, the primary contribution came from knee extensor strength, with the secondary contribution from knee extensor hypertonia and strength of hip flexors. In the swing, the primary contribution came from knee extensor hypertonia, with the secondary contribution from passive knee flexion ROM.

## Figures and Tables

**Table 1:** Impairment outcome measures (MMT, HYP, SMC, CON measures) involved in knee flexion in terminal stance, pre-swing and initial swing (K3–K5).

Impairment	Muscle Group	Outcome Measure
Weakness (MMT)	Hip flexors Knee extensors	MMT_HIP_FL MMT_KNEE_EXT
Hypertonia (HYP)	Knee extensors Knee flexors Plantar flexors	HYP_KNEE_EXT HYP_KNEE_FL HYP_PF
Lack of selectivity (SMC)	Hip flexors Knee extension	SMC_HIP_FL SMC_KNEE_EXT
Restricted range of motion (ROM)	Knee extensors	CON_KNEE_EXT

MMT: manual muscle testing; HYP: muscle hypertonia; SMC: selective motor control; ROM: range of motion; HIP_FL: hip flexors; KNEE_EXT: knee extensors; KNEE_FL: knee extensors; PF: plantar flexors; CON: contraction.

**Table 2:** Multiple linear regression analysis with selected knee ROM and angular velocity at toe-off as independent variables (*n* = 262).

	β	95% CI	*p*-Value
K5–K3			
HYP_KNEE_EXT	−5.75	[−8.74; −2.76]	0.0002
MMT_KNEE_EXT	2.76	[0.08; 5.44]	0.0433
Adjusted *R*^2^ = 0.0801, *F* = 11.0963, *p* < 0.0001			
K4–K3			<0.0001
HYP_KNEE_EXT	−2.74	[−4.98; −0.49]	0.0170
MMT_HIP_FL	2.01	[−3.89; −0.14]	0.0353
MMT_KNEE_EXT	4.04	[1.87; 6.21]	0.0003
Adjusted *R*^2^ = 0.0744, *F* = 7.2135, *p* < 0.0001			
K5–K4			
CON_KNEE_EXT	0.16	[0.01; 0.31]	0.0400
HYP_KNEE_EXT	−2.55	[−4.96; −0.14]	0.0384
SMC_HIP_FL	2.02	[1.56; −1.05]	0.1962
SMC_KNEE_EXT	−2.42	[2.03; −6.43]	0.2353
Adjusted *R*^2^ = 0.0398, *F* = 3.4010, *p* = 0.01			
V			
HYP_ KNEE_EXT	−39.86	[−60.60; −19.11]	0.0002
SMC_HIP_FL	39.68	[15.27; 64.09]	0.0016
Adjusted *R*^2^ = 0.1152, *F* = 16.0988, *p* < 0.0001			

β: slope of regression; CI: confidence interval at 95%; V: angular velocity at toe-off.

## Data Availability

The datasets generated during and/or analyzed during the current study are available from the corresponding author on reasonable request.
